# Efficient and mechanically robust stretchable organic light-emitting devices by a laser-programmable buckling process

**DOI:** 10.1038/ncomms11573

**Published:** 2016-05-17

**Authors:** Da Yin, Jing Feng, Rui Ma, Yue-Feng Liu, Yong-Lai Zhang, Xu-Lin Zhang, Yan-Gang Bi, Qi-Dai Chen, Hong-Bo Sun

**Affiliations:** 1 State Key Laboratory on Integrated Optoelectronics, College of Electronic Science and Engineering, Jilin University, 2699 Qianjin Street, Changchun 130012, China; 2 College of Physics, Jilin University, 2699 Qianjin Street, Changchun 130012, China

## Abstract

Stretchable organic light-emitting devices are becoming increasingly important in the fast-growing fields of wearable displays, biomedical devices and health-monitoring technology. Although highly stretchable devices have been demonstrated, their luminous efficiency and mechanical stability remain impractical for the purposes of real-life applications. This is due to significant challenges arising from the high strain-induced limitations on the structure design of the device, the materials used and the difficulty of controlling the stretch-release process. Here we have developed a laser-programmable buckling process to overcome these obstacles and realize a highly stretchable organic light-emitting diode with unprecedented efficiency and mechanical robustness. The strained device luminous efficiency −70 cd A^−1^ under 70% strain - is the largest to date and the device can accommodate 100% strain while exhibiting only small fluctuations in performance over 15,000 stretch-release cycles. This work paves the way towards fully stretchable organic light-emitting diodes that can be used in wearable electronic devices.

## Abstract (web summary)

Highly stretchable organic light-emitting diodes tend to suffer from a lack of mechanical robustness. Here, Yin *et al*. fabricate ordered buckled films by laying flexible light-emitting diodes on laser-ablated, prestretched substrates. The devices exhibit good emission stability over 15,000 stretching cycles.

## Graphical abstract

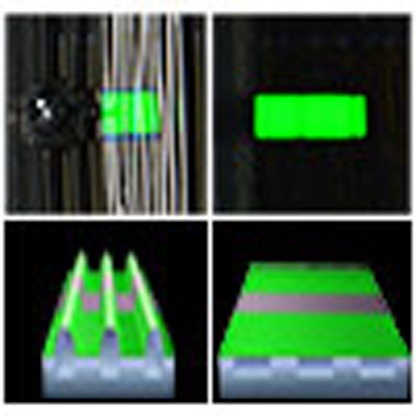

Efforts to create stretchable organic light-emitting devices (OLEDs) have been stimulated by rapid advances in flexible and stretchable electronics and optoelectronics^1^,^2^,^3^,^4^,^5^,^6^,^7^,^8^,^9^. Because stretchable OLEDs conform to any surface topology and are mechanically insensitive to fatigue strain, they have an increasingly important role in emerging applications such as deformable displays and electronic skin^10^,^11^,^12^,^13^,^14^,^15^,^16^. Several methods have been used to develop stretchable OLEDs. Stretchable electrical interconnects have been combined with discrete rigid light-emitting units to form a hybrid structure, which itself is not stretchable^10^. Intrinsically stretchable OLEDs have been created in which the constituent layers are stretchable and the component materials must be highly elastic^11^,^12^,^14^,^16^. Attaching an ultrathin film to an elastomeric substrate results in spontaneous buckling of the film, thus provides another approach for creating stretchable OLEDs. This strategy has been used to fabricate stretchable polymer-based OLEDs and photovoltaic devices on ultrathin foil substrates that can be stretched up to 100% of its original size^13^,^17^. Sufficient stretchability (tensile strain), luminous efficiency and mechanical robustness are crucial for the successful application of stretchable OLEDs. Although highly stretchable OLEDs have been reported, their application has been hindered by their low efficiency and insufficient mechanical robustness. The need to avoid physical and electrical damage to the devices under large levels of strain imposes limits on the structural design, material selection and controllability of the stretch-release process^18^,^19^,^20^. Simultaneous achievement of both mechanical robustness and high electroluminescent (EL) performance remains one of the most difficult challenges.

The reported polymer-based stretchable OLEDs on ultrathin PET foil substrate with buckling profile exhibited a highest luminance of 122 cd m^−2^ and efficiency of 0.17±0.06 cd A^−1^, and their mechanical robustness was not described^13^. A very thick active layer (200–300 nm) is needed due to the rough surface of the PET with a root mean square surface roughness of 26 nm and peak height of 700 nm, which results in the poor performance of the OLEDs. Moreover, the ultrathin OLEDs with uncontrolled and disordered buckling are easily misshapen and difficult to handle, which results in insufficient mechanical robustness and hampers their long-term stability. The best performance ever reported for the fully stretchable OLEDs is from the intrinsically stretchable devices based on a polymer light-emitting electrochemical cell, in which the highest efficiency is 11.4 cd A^−1^ at a peak brightness of 2,200 cd m^−2^, and it could survive 1,000 stretch-release cycles^14^. The necessity for the elasticity of each constituent layer limited selection of the materials and thereafter the luminous efficiency of this fully stretchable OLED. The characteristics reported thus far are still far below the requirement for the commercial application of OLEDs in displays and lighting.

In this work, we demonstrate high efficiency and mechanical robustness for highly stretchable OLEDs. We developed a laser-programmable buckling process to obtain stretchable OLEDs with an ordered-buckling profile, which not only creates options for the design and fabrication of the device but also allows the stretch-release cycles to be controlled. We achieved maximum efficiency of 72.5, 68.5 and 70 cd A^−1^ at mechanical strain of 0%, 40% and 70%, respectively. The OLEDs can accommodate tensile strain up to 100% and exhibit only small fluctuations in performance over 15,000 stretch-release cycles. To the best of our knowledge, our stretchable OLEDs have the highest efficiency and mechanical robustness reported to date. This simple and low-cost method is compatible with most OLED fabrication processes, such as spin-coating, thermal evaporation and ink-jet printing, and is independent of the OLED's component materials and structure. As a result, the method can be applied to any monochromatic or white OLEDs with complex structure to achieve even higher efficiencies.

## Results

### Stretchable OLEDs with an ordered-buckling profile

**Figure 1:**
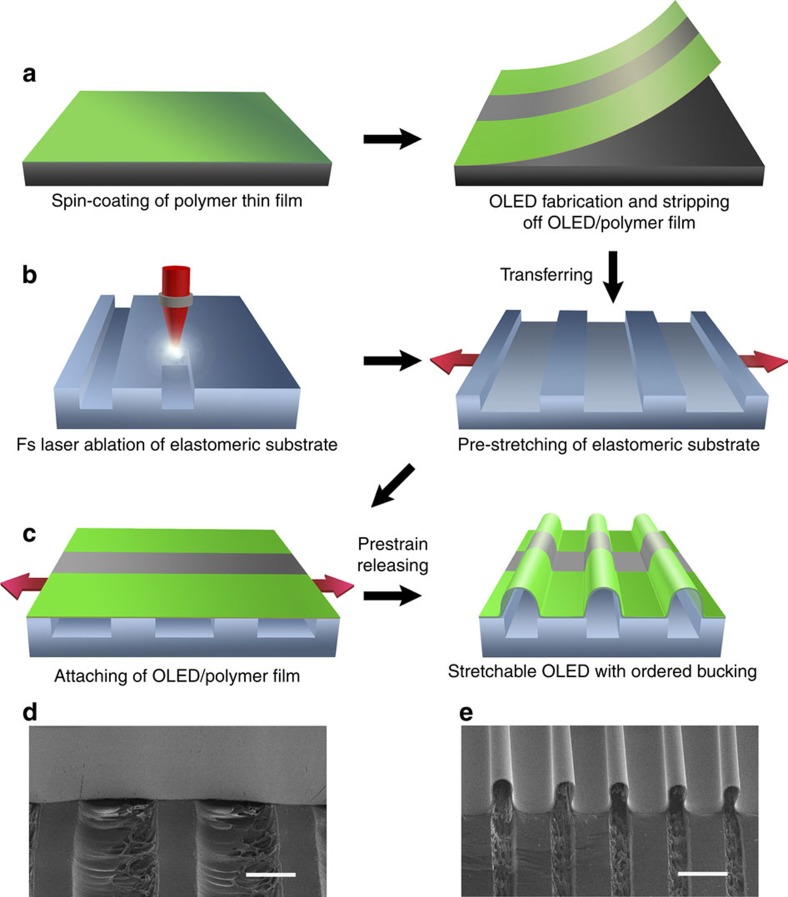
Schematic illustration of the fabrication process of stretchable OLEDs. (**a**) OLED preparation: spin-coating an ultrathin polymer film onto the silicon slice, followed by ultraviolet curing, and fabricating OLEDs on the ultrathin polymer film and then peeling off the OLED/polymer film from the Si substrate. (**b**) Substrate preparation: laser ablation of long-period gratings on the elastomeric substrate, and prestretching the grated elastomeric substrate. (**c**) Stretchable OLED assembly: attaching the OLED/polymer film onto the prestretched elastomeric substrate, and releasing the prestrains and obtaining OLEDs with ordered buckles. (**d**,**e**) 45°-tilted-view SEM images of the stretchable OLEDs corresponding to the steps of the fabrication process shown in **c**. Scale bars, 500 μm.

An ultrathin polymer film with a thickness of around 10 μm was fabricated by spin-coating a photosensitive prepolymer onto a pretreated Si substrate followed by UV curing (Fig. 1a, left). The small molecule-based top-emitting OLEDs were deposited by thermal evaporation onto the ultrathin polymer film. The OLED/polymer film was then peeled off from the Si substrate (Fig. 1a, right and Supplementary Fig. 1). We used femtosecond (fs) laser ablation to fabricate one-dimensional (1D) long-period gratings with programmable parameters on the surface of an elastomeric substrate (Fig. 1b, left). A grating period of 570 μm on an area as large as 4 cm × 4 cm can easily be achieved (Supplementary Fig. 2). The depth of the grooves is dependent on the fs laser fluence (Supplementary Fig. 3). The elastomeric substrate with defined groove width and depth was then prestretched uniaxially with 120% strain (Fig. 1b, right). The peeled-off OLED/polymer film was transferred and attached to the prestretched elastomeric substrate (Fig. 1c, left). The OLED/polymer film adhered to the top surface of the grating lines and was suspended above the grating grooves. Releasing the strain on the prestretched elastomeric substrate thus resulted in the OLED buckling above the grooves (Fig. 1c, right). We obtained stretchable OLEDs with ordered buckling that exhibited the same period as that of the 1D gratings on the elastomeric substrate, which is confirmed by the tilted-view scanning electron microscopic (SEM) images (Fig. 1d,e).

The strain applied to the prestretched elastomeric substrate results in variations in the grating parameters, which we examined under 120% strain. Compared with an elastomeric substrate under no strain, the groove depth decreased from about 110 to 90 μm, which indicates that large strain results in a small variation in groove depth, while the thickness of the stretched elastomeric substrate was wholly decreased (Supplementary Fig. 4). Large groove depth contributes to the suspension of the OLEDs above the grooves. The grating period increased from 570 to 1,250 μm. The widths of the grooves and lines increased from 170 and 400 μm to 800 and 450 μm, respectively, which correspond to a strain of 370% for the grooves and 12.5% for the lines. The compressive strain on the grating grooves is therefore much larger than that on the grating lines after releasing the strain. This difference is important for the formation of ordered buckling only above the grooves once the OLEDs are attached to the elastomeric substrate. We noted that the edges of the grooves were uniform and neat without unnecessary molten trace, which is a benefit of the high precision and negligible thermal diffusion of the fs laser ablation process^21^.

### Device stretchability

**Figure 2:**
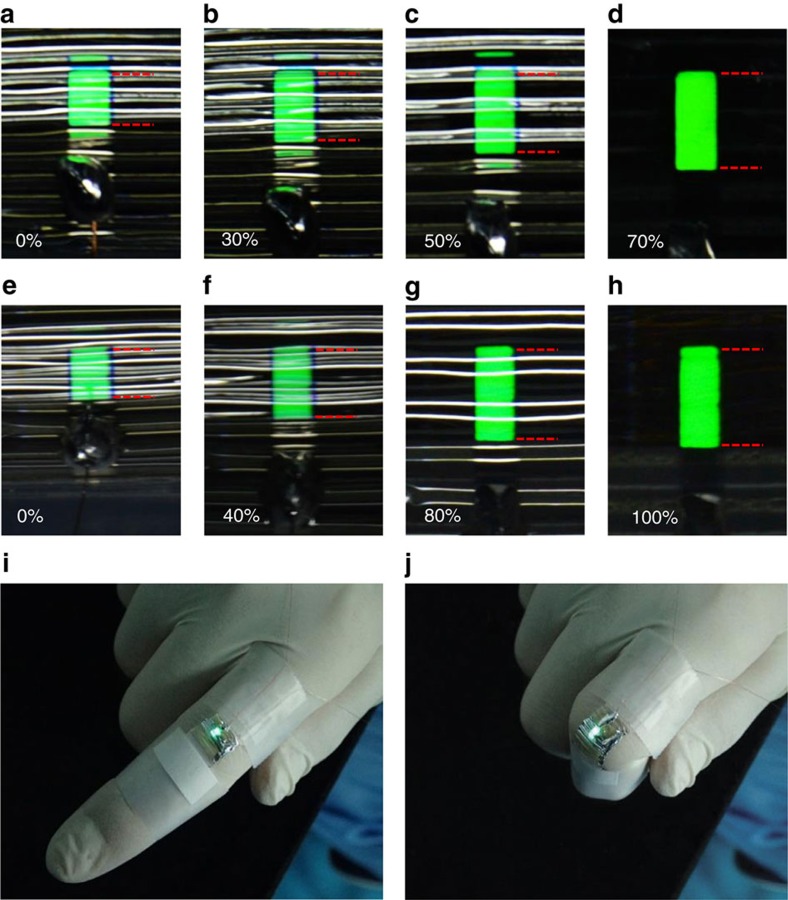
Demonstrations of the OLED stretchability. Photographs of the stretchable OLEDs based on a 120% prestrained substrate at 5 V with strain values of 0% (**a**), 30% (**b**), 50% (**c**) and 70% (**d**). Photographs of the stretchable OLEDs based on a 200% prestrained substrate at 5 V with strain values of 0% (**e**), 40% (**f**), 80% (**g**) and 100% (**h**), and mounted on an extended (**i**) and bent (**j**) finger joint. The green area corresponds to the green light emission from the operating OLEDs. The emission area of the fully stretched OLED (**d**,**h**) is 1.5 × 3.5 mm^2^. The dashed red line defines the edge of the emitting devices, which is blurred due to the reflection of the buckling profile.

We used thermal evaporation to fabricate small molecule-based OLEDs using tris(2-phenylpyridine)iridium(III) (Ir(ppy)_3_) as an emitter. Uniform and bright emission across the entire luminous area was observed for these stretchable OLEDs under various strain levels and at a driving voltage of 5 V. With a 120% prestrain of the elastomeric substrate, we achieved stretchable OLEDs with a maximum tensile strain of 70% (Fig. 2a–d). Periodic buckles were formed after releasing the prestrain, which returned the stretchable OLEDs to a state that corresponded to 0% tensile strain. In this case, the bending radius for the area of the OLEDs that is suspended above the grooves is about 100 μm. The high flexibility of the ultrathin polymer film permits such a small bending radius^22^,^23^,^24^. We should note that the tensile strain corresponds to the stretchability of the whole OLED/polymer film/elastomeric substrate system. The buckling profile of the OLED formed at the initial state of 0% tensile strain, which creates certain compressive stress inside the OLED structure. The period of the ordered buckles increased uniformly with increasing tensile strain, and the bending radius also increased, while the stress inside the OLED structure is decreased. Under a tensile strain of 40%, the bending radius for the area of the OLEDs that was suspended above the grooves increased to around 400−600 μm (Supplementary Fig. 5). At the maximum strain value of 70%, the buckles disappeared and the OLEDs became planar. As the strain decreased gradually to 0% (the unstrained state), the periodic buckling formed again. Repeated stretch-release cycles were conducted, during which the stretched devices displayed uniform and bright emission across the entire luminous area (Supplementary Movie 1). The stretchability of the OLEDs can be improved further by increasing the prestrain value. For example, the maximum tensile strain can be increased to 100% by increasing the prestrain to 200% (Fig. 2e–h). We demonstrated the potential use of these stretchable OLEDs for wearable electronics by mounting the OLED on the back of a finger joint (Fig. 2i,j). When the finger was bent, the strain value is about 55–60% for the stretchable and foldable OLED. When the finger was repeatedly bent, the buckles of the OLED remained periodic and ordered (Supplementary Movie 2).

### EL performance of the stretchable OLEDs

**Figure 3:**
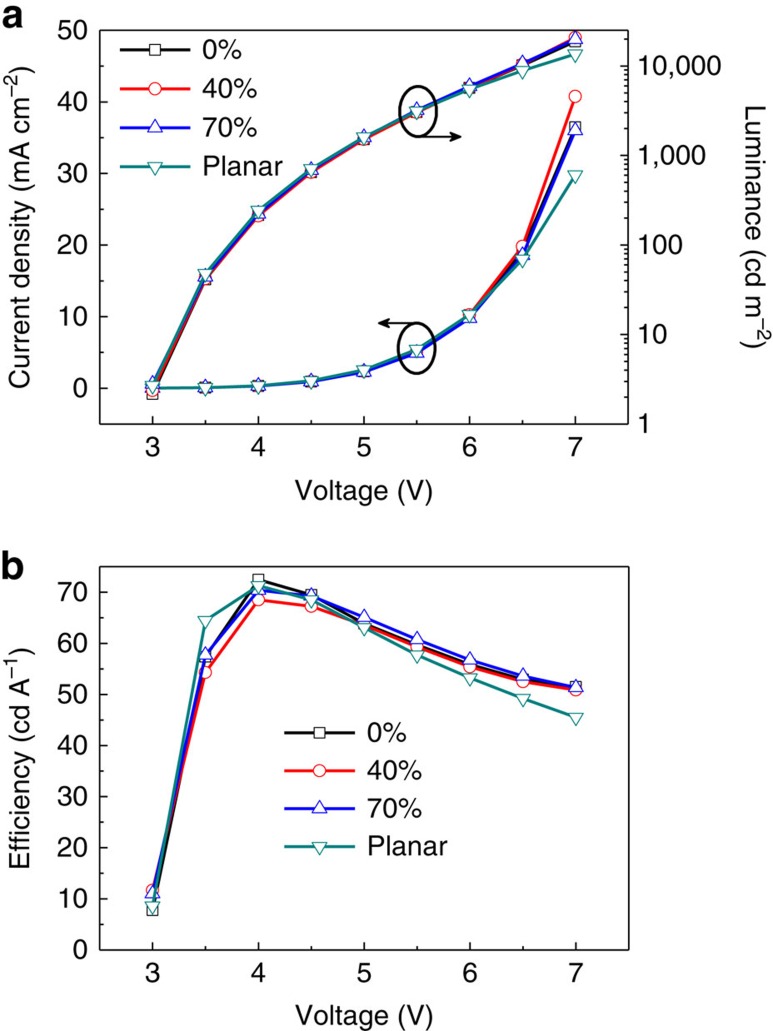
EL performance of the stretchable OLEDs. Current density–luminance–driving voltage characteristics (**a**) and current efficiency–driving voltage characteristics (**b**) of stretchable and planar devices.

We fabricated the OLED on the polymer/Si substrate, and then the OLED/polymer film was stripped off entirely from the Si substrate and transferred to the prestretched elastomeric substrate. This fabrication process permits flexibility in the selection of the materials and fabrication process used for the stretchable OLEDs, which benefits its EL performance. In this work, we fabricate small molecule-based stretchable OLEDs with highly efficient phosphorescent emitter of (Ir(ppy)_3_) by using thermal evaporation method. Moreover, the polymer film as their substrates spin-coated on the ultrasmooth Si slices has an ultrasmooth surface with a root mean square roughness of 0.35 nm (Supplementary Fig. 6), which provides the option design of the device structure compared with the rough PET foil substrate^13^ and has further beneficial effect on the high efficiency of the OLEDs. We examined the current density–luminance–voltage and efficiency–voltage characteristics of the stretchable OLEDs using 120% prestrain and 70% maximal tensile strain (Fig. 3). Planar OLEDs on the Si substrate (before removal from the Si substrate) were also examined for comparison. The planar OLEDs turn on at 3 V. The current efficiency increases rapidly with the driving voltage, and reaches 71 cd A^−1^ at 4 V with a brightness of 243 cd m^−2^. Under various levels of tensile strain, the stretchable OLEDs show comparable EL performance to that of the planar devices and the same turn-on voltage of 3 V. At 4 V, the maximal efficiency is 72.5, 68.5 and 70 cd A^−1^ at strain of 0%, 40% and 70%, respectively. The stretchable OLEDs described here are clearly superior to previously reported stretchable OLEDs in both luminance and efficiency.

### Mechanical robustness of the stretchable OLEDs

The mechanical stability of stretchable OLEDs is very important for their application. We examined their mechanical robustness by comparing the luminance and current efficiency at a driving voltage of 5 V under various strain values from 0 to 70%. The EL performance of the stretchable OLED at different strain values on the OLED regions both on the grating lines and on the grating grooves were measured to verify the effect of the film stretching on the light collection (Supplementary Figs 7–9 and Supplementary Note 1). As can be seen, both brightness and efficiency are comparable to each other and it is slightly lower for the OLED region on the grooves. Moreover, they both exhibited slight variations at different strain values. The variations are 3.5% and 4.2% for the luminance, and 2.7% and 5.6% for the efficiency from the grating line and groove regions, respectively. Furthermore, the characteristics of more focused areas on both grating line and large viewing angle regions, and zoomed-out area with a large emitting region for the average luminance as a function of strain are also measured (Supplementary Figs 10–13 and Supplementary Notes 2 and 3).

**Figure 4:**
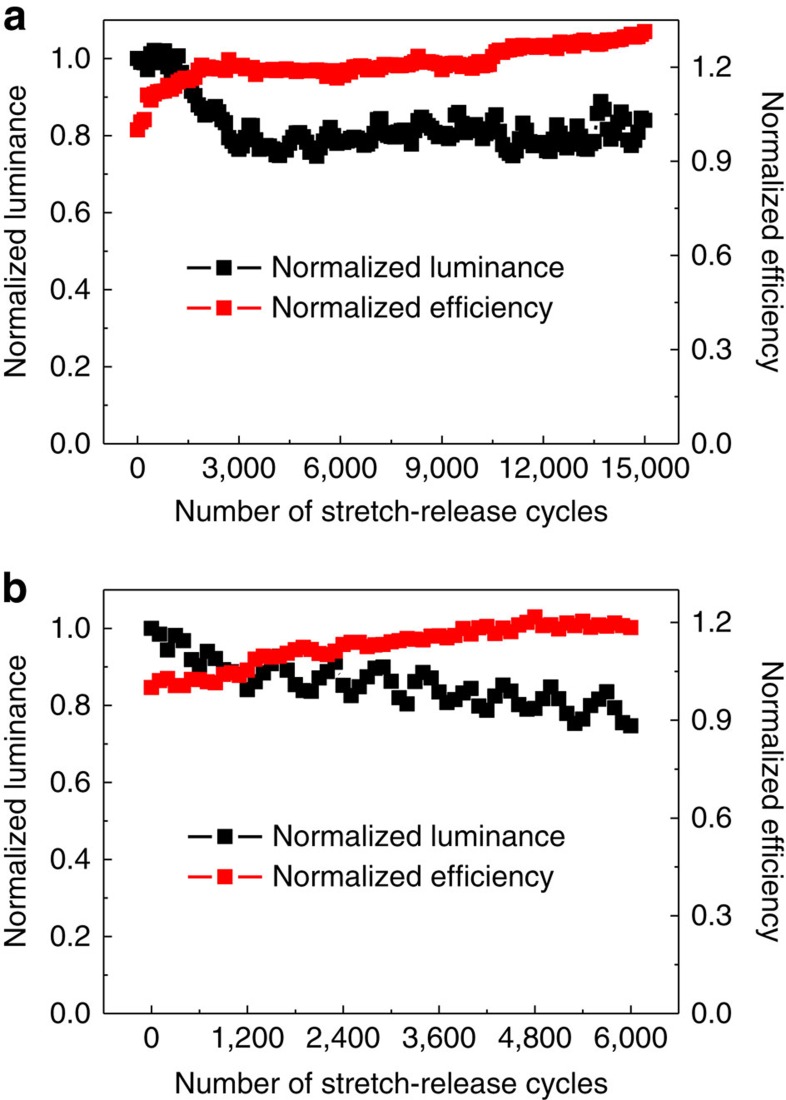
Mechanical robustness characterization of the stretchable OLEDs. Normalized luminance and current efficiency vs the number of stretch-release cycles for the stretchable OLEDs at 5 V between 0 and 20% (**a**) and 0% and 40% (**b**) strain.

The mechanical robustness was further examined by comparing their EL performance under repeated stretch-release cycles (Fig. 4). In this case, the EL performance was measured at 0% strain state during the stretch-release cycles. At strain values between 0 and 20%, luminance degraded only around 16% within the 15,000 stretch-release cycles (Fig. 4a). At strain values between 0 and 40% (Fig. 4b), luminance degraded by 25% within 6,000 cycles. By contrast, current efficiency slightly increased with increasing number of stretch-release cycles. This can be attributed to the decreased current density (Supplementary Fig. 14). These results show that stretchable OLEDs with an ordered buckling profile are mechanically robust. The buckles were uniformly stretched with increasing strain and returned to their original profile once the strain was removed. The OLED could therefore retain the ordered buckling profile and avoid both variations in the surface texture of the functional layers and the sheet resistance of the composite electrodes during the stretch-release cycles, which collectively contributed to its mechanical robustness.

### Tunability of the device stretchability

**Figure 5:**
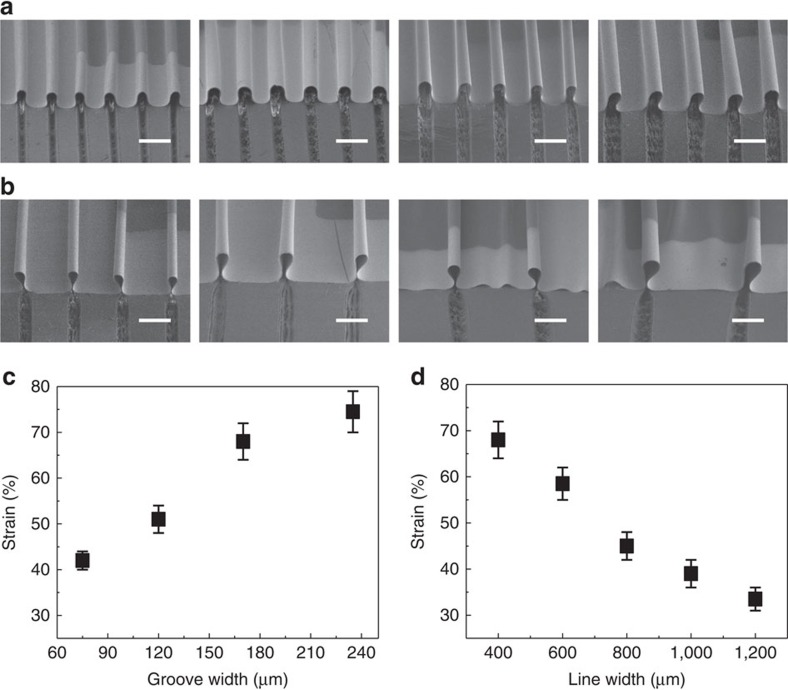
Tunable stretchability of the stretchable OLEDs. 45°-tilted-view SEM images of the stretchable OLEDs on the 1D corrugated elastomeric substrate with a fixed grating line width of 400 μm and varied grating groove width of 75, 120, 170 and 235 μm from left to right (**a**), and a fixed grating groove width of 170 μm and varied grating line width of 600, 800, 1,000 and 1,200 μm from left to right (**b**). Scale bars, 500 μm. (**c**) The relationship between the maximal strain of the stretchable OLEDs and the groove width of the gratings (the line width is fixed at 400 μm). (**d**) The relationship between the maximal strain of the stretchable OLEDs and the line width of the gratings (the groove width is fixed at 170 μm). Error bars represent the s.e.m. from three independent samples (*n*=3).

The stretchability of the OLEDs can be modified by tuning the programmable parameters of the 1D gratings on the elastomeric substrate. We investigated the dependence of stretchability on the width of the lines and grooves (Fig. 5). The maximum strain of the stretchable OLED increases with increasing groove width at a fixed line width of 400 μm (Fig. 5a,c and Supplementary Fig. 15 a–d). By contrast, the maximum strain decreases with increasing line width at a fixed groove width of 170 μm (Fig. 5b,d and Supplementary Fig. 15 e–h). By comparing the variations in the groove and line widths before and after stretching (Supplementary Fig. 16), we can conclude that the strain is imposed mainly on the grating grooves where buckles rise up. The lines holding the inelastic polymer substrates have nearly no contraction after the strain is released, except for lines wider than 1,000 μm, where a few small buckles can be observed (Fig. 5b). The laser-programmable buckling process for the stretchable OLEDs allows the stretchability to be tuned and the stretch-release cycles to be controlled. We should note that appropriate grating parameters are necessary for obtaining ordered buckles and thereafter highly stretchable and mechanically robust OLEDs. (Supplementary Fig. 17 and Supplementary Note 4).

## Discussion

In summary, we have developed highly stretchable OLEDs with unprecedented levels of efficiency and mechanical robustness. A laser-programmable buckling process was used to create ordered buckles on the OLEDs, which permits controllable stretch-release cycles and contributes to the high stretchability and excellent mechanical robustness of the devices. Owing to its independence of the materials used and the structure of the device, the method described here creates options for the design and fabrication of stretchable OLEDs and high efficiency is easily achievable. This is an important step towards producing fully stretchable OLEDs for commercial applications in wearable electronics, especially in lighting and display applications where high efficiency and mechanical stability are required.

## Methods

### Long-period grating fabrication on the elastomeric substrate

Long-period gratings were fabricated on the elastomeric substrate (3M VHB 4905) using the fs laser-programmable ablation process with focused fs laser pulses (800 nm peak wavelength, 100 fs pulse width and 1,000 Hz repetition rate) (Solstice, Spectra-Physics). The 3M VHB 4905 tape is an adhesive elastomer with very high viscosity confirmed by dynamic adhesion performance test, such as 90° peel adhesion of 21 N cm^−1^, normal tensile of 690 kPa and dynamic overlap shear of 480 kPa (obtained from www.3M.com). The grating grooves were ablated on the 3M VHB 4905 tape by the focused fs laser pulse, while the surface of the grating lines was not treated by the laser, so that the high viscosity can be maintained. The high viscosity permits the strong and durable bonding between the ultrathin OLEDs and the top surface of the grating lines. The process was performed on a custom-made two-dimensional moving stage controlled by a user-defined direct-write program. The laser scanning speed was 2 mm s^−1^. A laser fluence of 6,000 W cm^−2^ was used to fabricate microstructures on the elastomers. A large patterned area of 4 cm × 4 cm can be fabricated with the same laser processing parameters in about 2 h (Supplementary Fig. 2).

### Fabrication of stretchable OLEDs

Si substrates were cleaned with acetone, ethanol and deionized water in sequence. After the silicon was dried and cooled to room temperature, an ultrathin layer of photoresist (NOA63, Norland Products, Inc.) was spin-coated onto the Si substrate at 6,500 r.p.m. for 30 s and then cured by ultraviolet exposure. The ultraviolet-cured polymer film on the Si substrate was loaded into a thermal evaporation chamber. The top-emitting OLEDs were fabricated on the polymer/Si substrate by thermal evaporation of the following materials in sequence: Ag as anode; MoO_3_ as anodic modification layer; N, N′ diphenyl-N, N′-bis (1,1′-biphenyl)-4,4′-diamine (NPB) as hole transporting layer; tris(2-phenylpyridine)iridium(III) (Ir(ppy)_3_) doped N, N′-dicarbazolyl-3,5-benzene (mCP) as emitting layer; 1,3,5-tris(*N*-phenyl-benzimidazol-2-yl)benzene (TPBi) as electron transporting layer; and Ca/Ag as cathode. The device structure is Ag (80 nm)/MoO_3_ (3 nm)/NPB (40 nm)/mCP:Ir(ppy)_3_ (6%, 20 nm)/TPBi (35 nm)/Ca (3 nm)/Ag (18 nm).

The cured polymer film precoated with the OLEDs was peeled off from the Si substrate and a freestanding OLED/polymer film was obtained (Supplementary Fig. 1). The patterned elastomeric substrate was stretched with a prestrain value of 120% (200% for the case shown in Fig. 2e–h) by a home-made moving stage. The freestanding OLED/polymer film was transferred to the surface of the prestretched elastomeric substrate, which is adhered to the grating lines and suspended on the grating grooves of the 1D gratings because of the large groove depth. Ordered buckles arose after releasing the prestrain. Electrical contact was made with copper wires and drops of eutectic gallium–indium (EGaIn) at the point where the anode and cathode of the OLEDs extended out of the emitting region of the devices. EGaIn is a commercially available metal alloy (75% Ga, 25% In by weight) with a melting point of 16 °C, which ensures it remains in a liquid state at room temperature^25^. The EGaIn drops are mouldable^26^ and conformed to the buckles on the ultrathin devices and changed shape with the buckles when the devices were stretched. EGaIn has low resistivity (29.4 × 10^−6^ Ω cm)^27^ and low toxicity, which make it suitable for use as a connection electrode in stretchable and wearable electronics.

### Characterization of stretchable OLEDs

The stretch-release tests were performed on a home-made moving stage. Photographs were taken by a Nikon digital single-lens reflex camera. SEM measurement was carried out on a JEOL JSM-7500F scanning electron microscope (JOEL Ltd.). Atom force microscopy (AFM) imaging was performed on a Dimension Icon AFM (Bruker Corporation). The film thickness was measured using an XP-2 stylus profilometer (Ambios Technology, Inc.). The current–luminance–voltage curves for the planar and stretchable OLEDs were measured with a Keithley 2400 source meter and a Photoresearch PR-655. Photoresearch PR-655 with MS-2.5 × MicroSpectar lens was employed for the luminance (in the unit of cd m^−2^) and EL spectra measurement, which collect light with a field coverage of 510 μm in diameter. The OLED/polymer film adhered to the top surface of the grating lines and was suspended above the grating grooves. Therefore, the stretchable OLED is buckled above the grooves with small bending radius. The OLED on the grating lines is also buckled due to the compressive strain, while its bending radius is larger than that above the grooves. The luminance and EL spectra measurement was performed on the OLED regions both on the grating lines and on the grating grooves to verify the effect of the film stretching on the light collection. They are shown in Supplementary Fig. 7 as black circle and white circle with a diameter of 510 μm. The characteristics of more focused areas on both grating line and large viewing angle regions, and zoomed-out area with a large emitting region for the average luminance as a function of strain are also measured. Photoresearch PR-705 with a tunable lens of MS-55 MacroSpectar was employed for these measurements. For the measurements of the focused areas, a 1/8° aperture was selected which collected light with a field coverage of 120 μm in diameter. For the measurements of average luminance, a 1° aperture was selected which collected light with a field coverage of 1,350 μm in diameter by tuning the MS-55 MacroSpectar lens. They are shown in Supplementary Fig. 10 as small (120 μm in diameter) and large (1,350 μm in diameter) circles. All measurements for the stretchable OLEDs were performed in air at room temperature without encapsulation.

### Data availability

The authors declare that the data supporting the findings of this study are available within the article and its Supplementary Information files.

## Additional information

**How to cite this article**: Yin, D. *et al*. Efficient and mechanically robust stretchable organic light-emitting devices by a laser-programmable buckling process. *Nat. Commun.* 7:11573 doi: 10.1038/ncomms11573 (2016).

## Supplementary Material

Supplementary Information Supplementary Figures 1-17 and Supplementary Notes 1-4

Supplementary Movie 1 Stretchable OLEDs undergoing repeated stretch-release cycles. Stretchable OLEDs are stretched repeatedly with strain values between 0% and 70% at room temperature in the air. The driving voltage is 5 V.

Supplementary Movie 2 Stretchable OLEDs mounted on the finger joint. A stretchable OLED is mounted on the back side of the finger joint. The device is stretched and bended repeatedly with the repeated finger bending. The driving voltage is 5 V.
