# Supplementary material for: Efficient and mechanically robust stretchable organic light-emitting devices by a laser-programmable buckling process
Source: Nat Commun. 2016 May 17;7:11573. doi: 10.1038/ncomms11573 (PMC4873666; doi:10.1038/ncomms11573)
Supplement: Supplementary Information — Supplementary Figures 1-17 and Supplementary Notes 1-4 [file ncomms11573-s1.pdf]

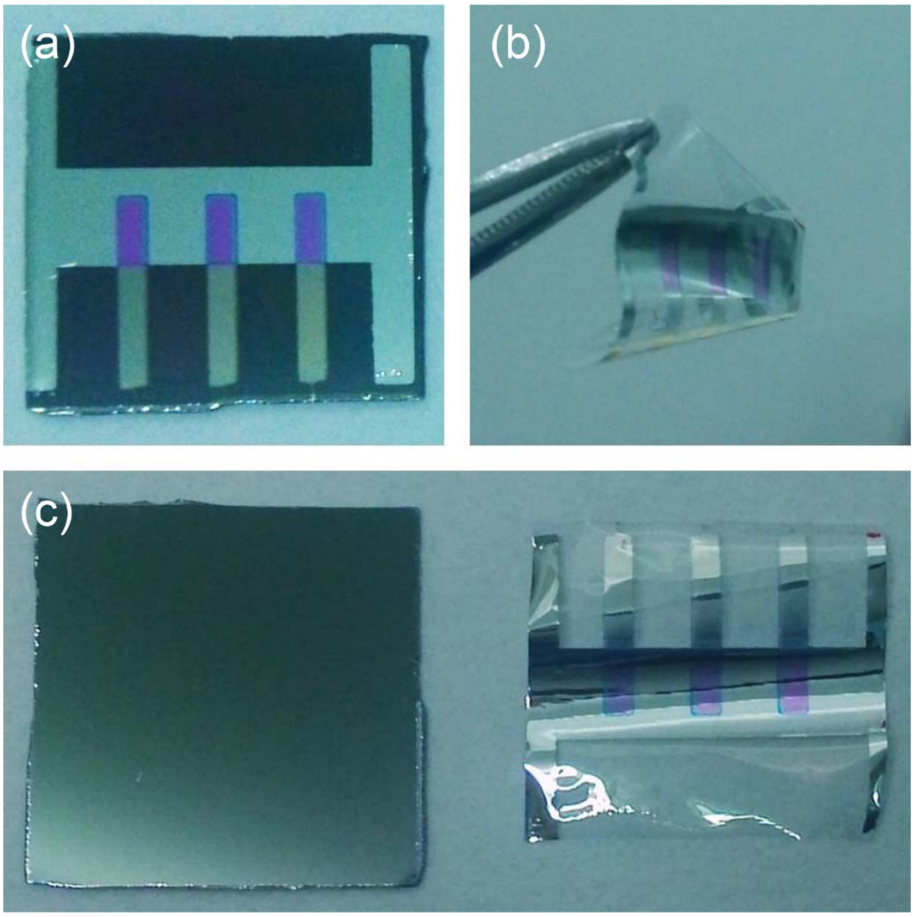

Supplementary Figure 1. OLEDs/polymer thin film before and after peeled off from silicon substrate. (a) OLEDs/polymer film fabricated on the Si substrate. (b) Free-standing OLEDs/polymer film peeled off from the Si substrate. (c) The Si substrate after peeling off of the OLEDs/polymer film. The ultrathin OLEDs could be peeled off from the Si substrate entirely.

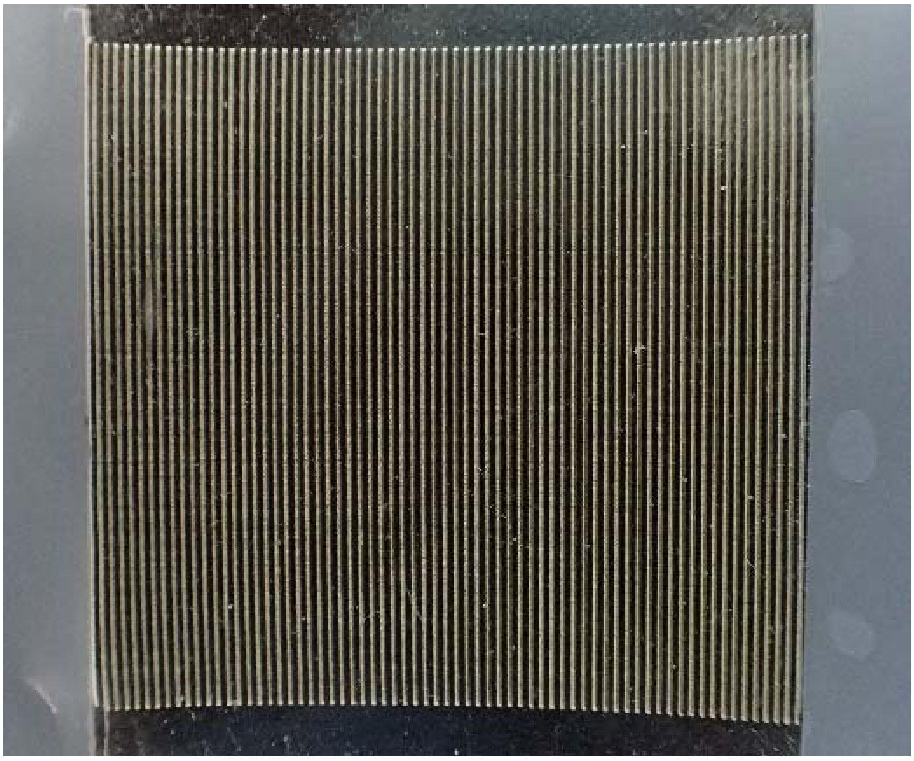

Supplementary Figure 2. Periodic grating on the elastomeric substrate by fs laser ablation process. Photograph of the 1-D long-period grating on elastomeric substrate with a period of 570 μm in a scale as large as 4 cm × 4 cm fabricated by Fs laser ablation process.

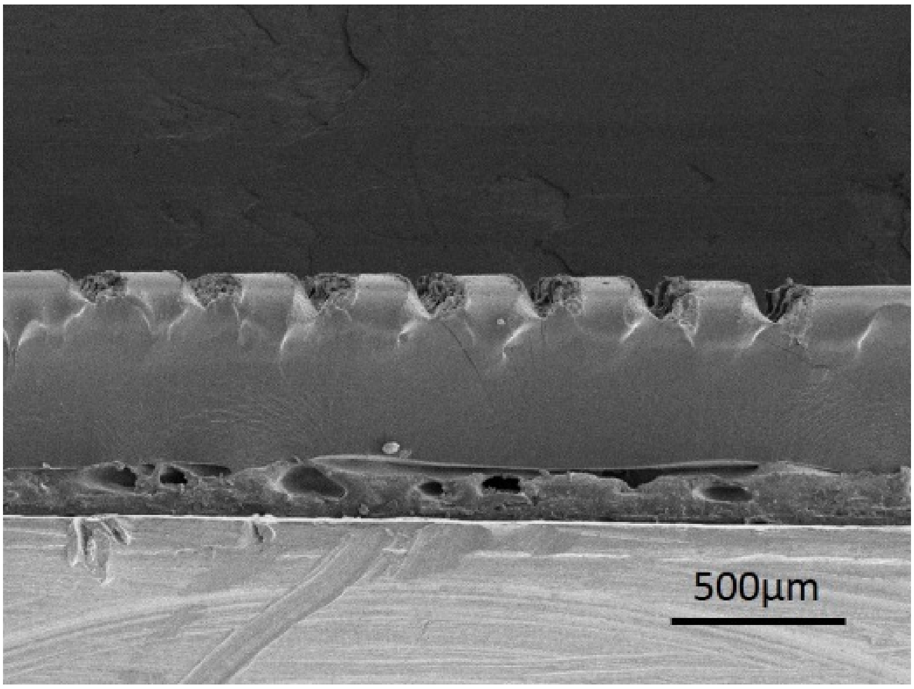

Supplementary Figure 3. Groove depth dependence on the laser fluence. Cross-sectional SEM image of gratings fabricated on elastomeric substrate with different Fs laser fluence. The depths of the grooves were 73µm, 79µm, 87µm, 97µm, 104µm, 108µm and 113 µm corresponding to the Fs laser fluence of 3000 W cm^{-2}, 3500 W cm^{-2}, 4000 W cm^{-2}, 4500 W cm^{-2}, 5000 W cm^{-2}, 5500 W cm^{-2} and 6000 W cm^{-2} from left to right, respectively.

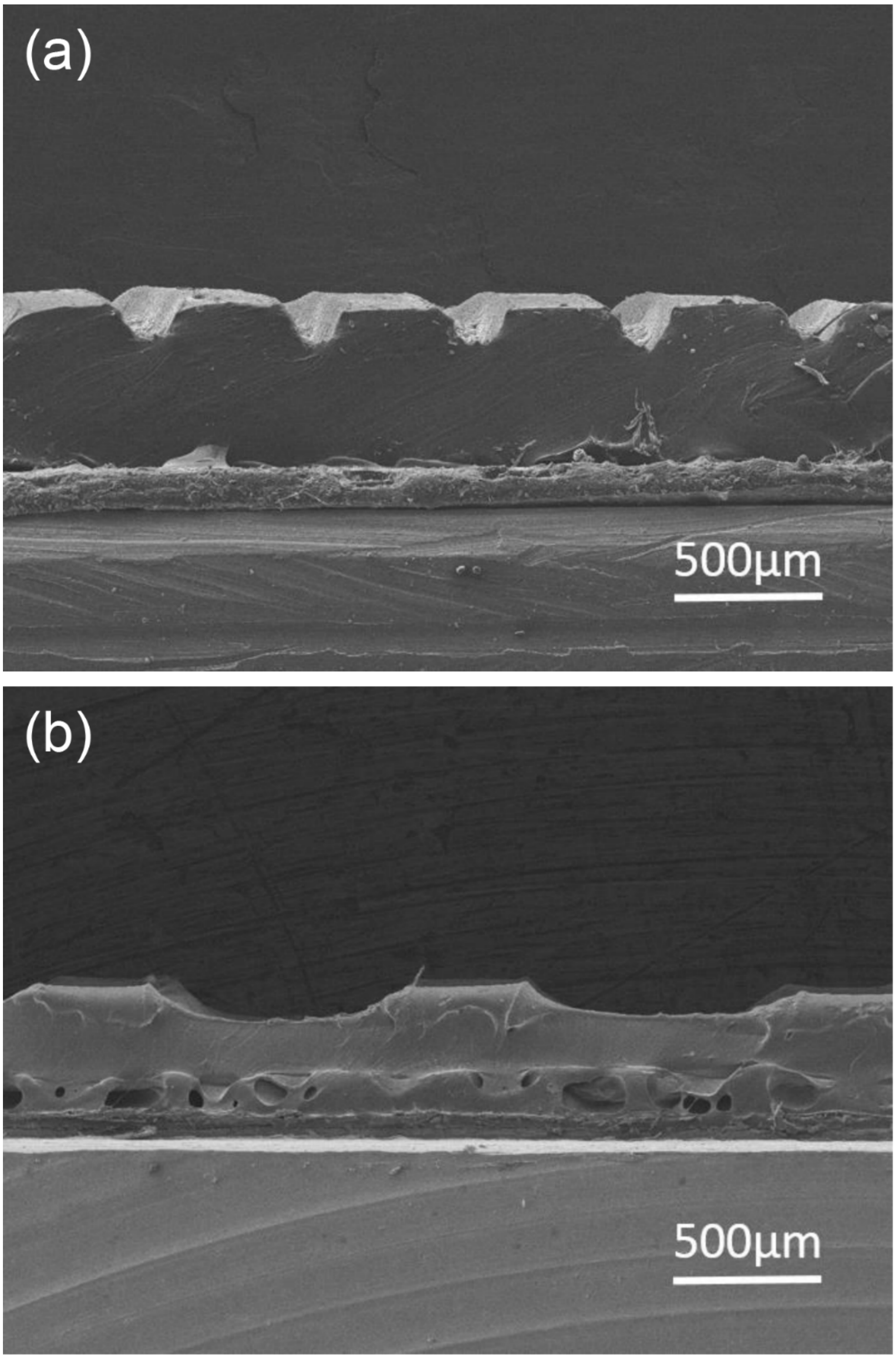

Supplementary Figure 4. Stretching the elastomeric substrate with the long-period gratings. Cross-sectional SEM images of gratings before (a) and after (b) stretching with 120% strain. The widths of grating lines and grooves are 400 μm and 170 μm in (a) and 450 μm and 800 μm in (b) respectively. The groove depths are about 110 μm and 90 μm before (a) and after (b) stretching respectively.

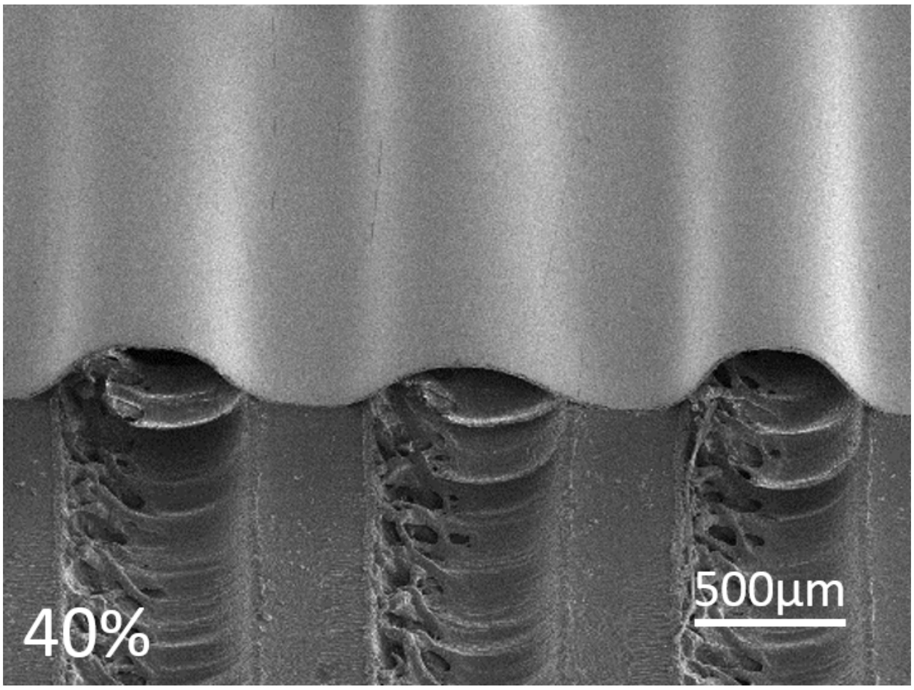

Supplementary Figure 5. Morphology of the stretchable OLEDs at 40% strain. SEM image of stretchable OLEDs at 40% strain. The bending radius of the ordered buckles is about 400µm–600 µm.

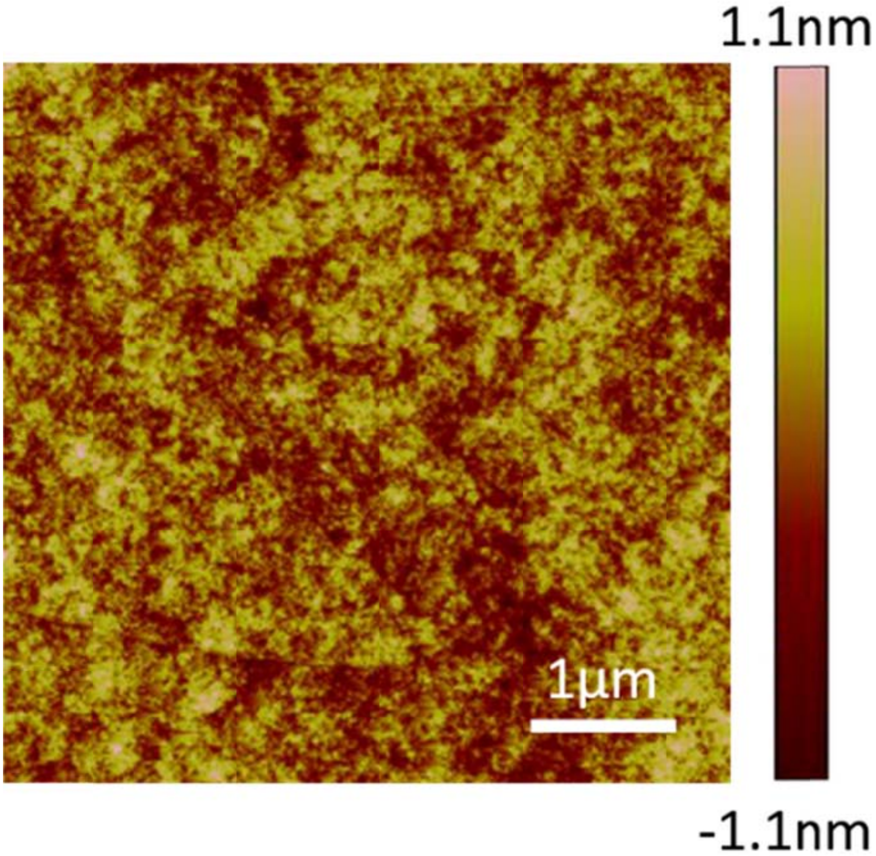

Supplementary Figure 6. Surface morphology of the ultrathin polymer substrate. AFM image of the ultrathin polymer film spin-coated on the Si substrate. The RMS roughness value of the ultrathin polymer substrate is 0.35 nm. The very smooth surface is suitable for fabricating highly efficient OLEDs.

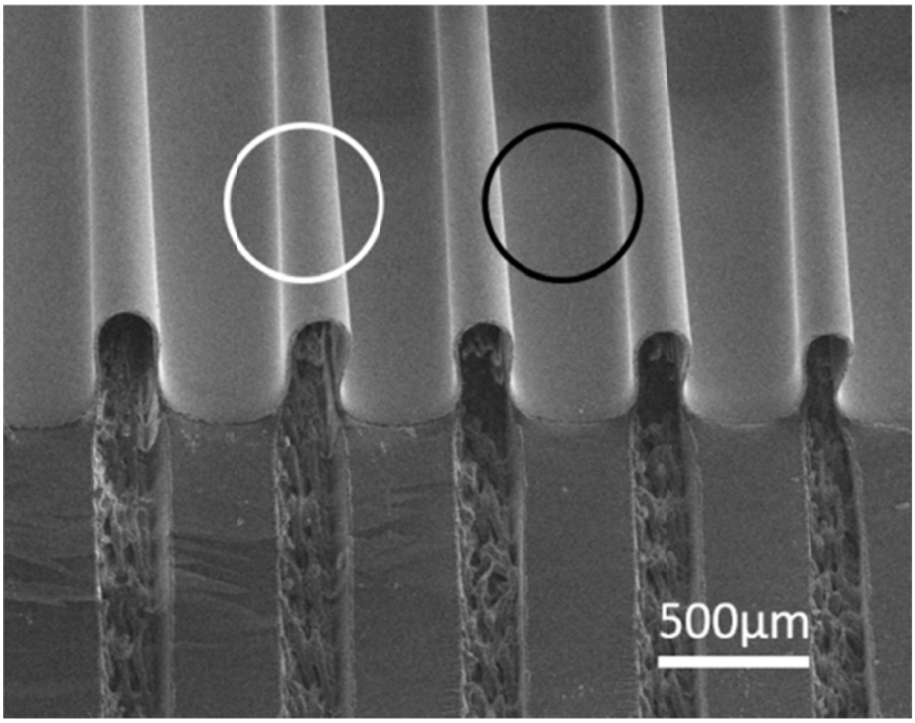

Supplementary Figure 7. SEM image of the stretchable OLEDs at 0% tensile strain. The circles with diameter of 510 µm correspond to the measured OLED region on the grating line (black circle) and on the grating groove (white circle). Photoresearch PR-655 with MS-2.5x MicroSpectar lens was employed for the luminance (in the unit of cd m^{-2}) and EL spectra measurement, which collect light with a field coverage of 510 µm in diameter. The luminance and EL spectra measurement was performed on the OLED region both on the grating lines and on the grating grooves to verify the effect of the film stretching on the light collection.

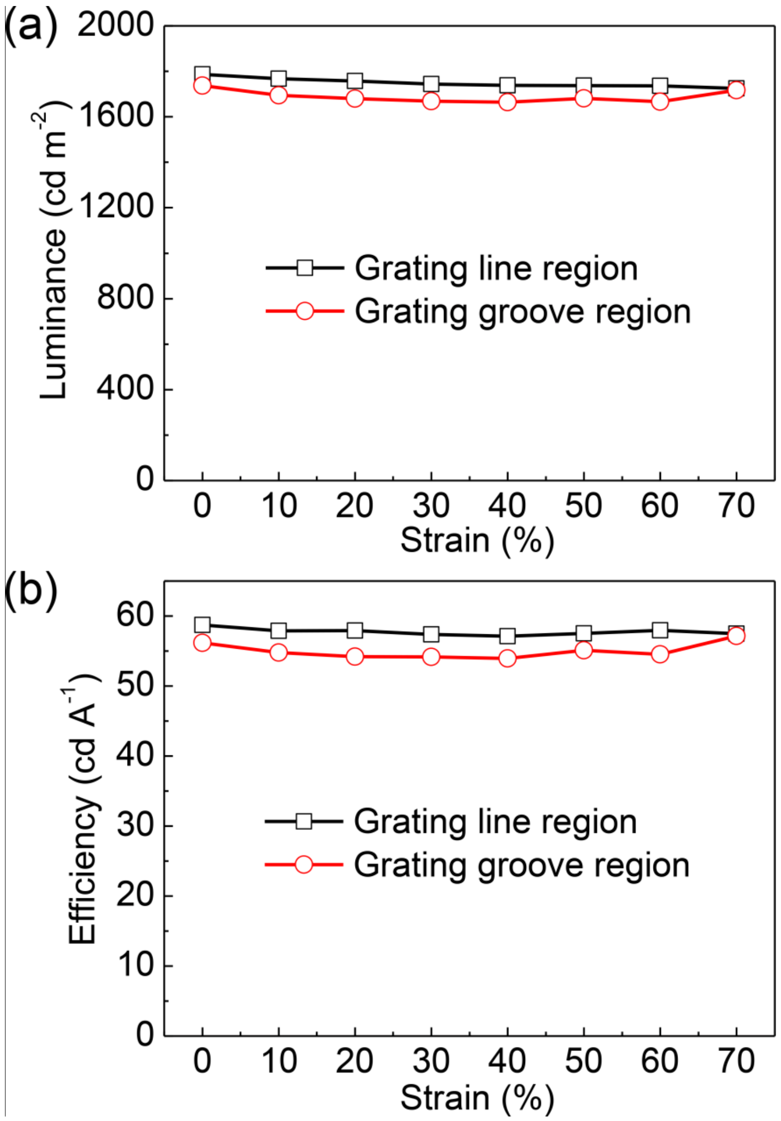

Supplementary Figure 8. **EL performance of the stretchable OLEDs at different region.** Luminance (a) and efficiency (b) measured from the OLED region on the grating lines (black) and on the grating grooves (red) vs strain values at a driving voltage of 5 V. The luminance measured from the OLED region on the grating lines and grooves are compared. As can be seen, both brightness and efficiency are comparable to each other and it is slightly lower for the OLED region on the grooves. Moreover, they both exhibited slight variations at different strain values. The variations are 3.5% and 4.2% for the luminance, and 2.7% and 5.6% for the efficiency from the grating line and groove regions, respectively, which can be attributed to the change of the profile during stretching.

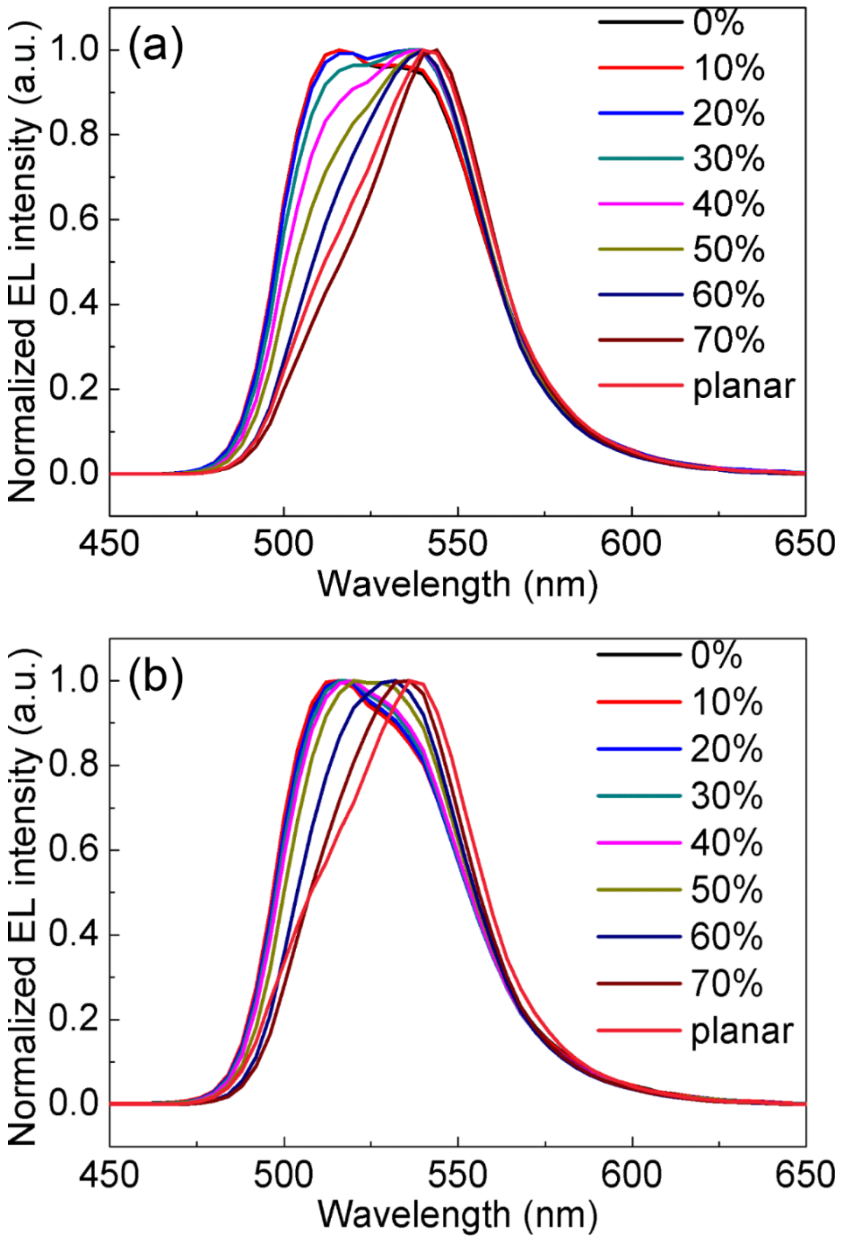

Supplementary Figure 9. EL spectra of the stretchable OLEDs. The normal-directional EL spectra measured from the OLED region on the grating lines (a) and on the grating grooves (b) at 5 V with different strain values. The normal-directional EL spectrum of a planar device is plotted for comparison.

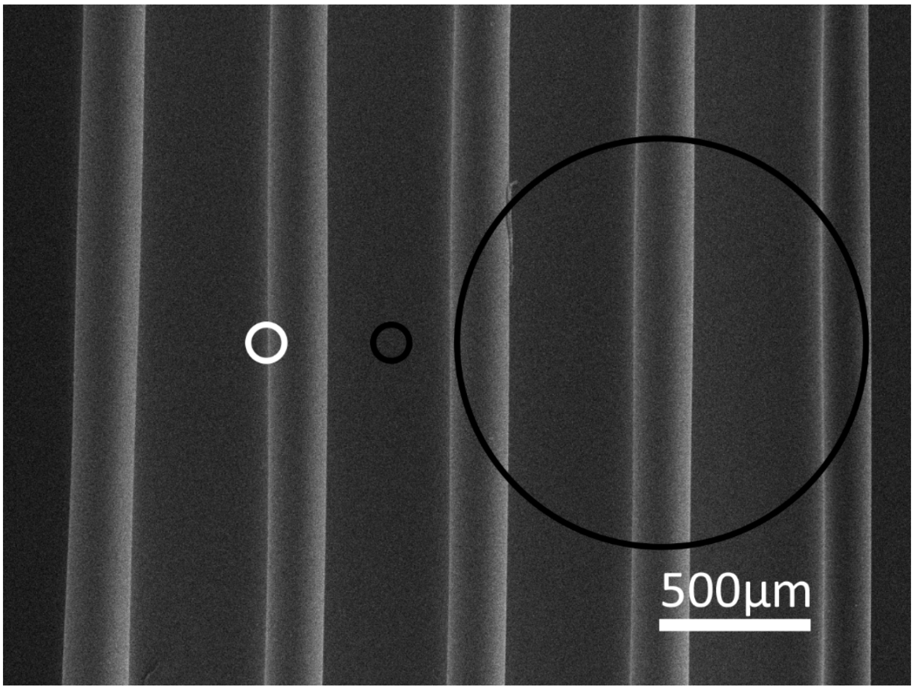

Supplementary Figure 10. SEM image of the stretchable OLEDs at 0% tensile strain. The small circles with diameter of 120 µm correspond to the measured OLED region focused on the grating line region (black circle) and large viewing angle region (white circle), respectively. The large circle with diameter of 1350 µm corresponds to the zoomed out region for the average luminance measurement (black circle).

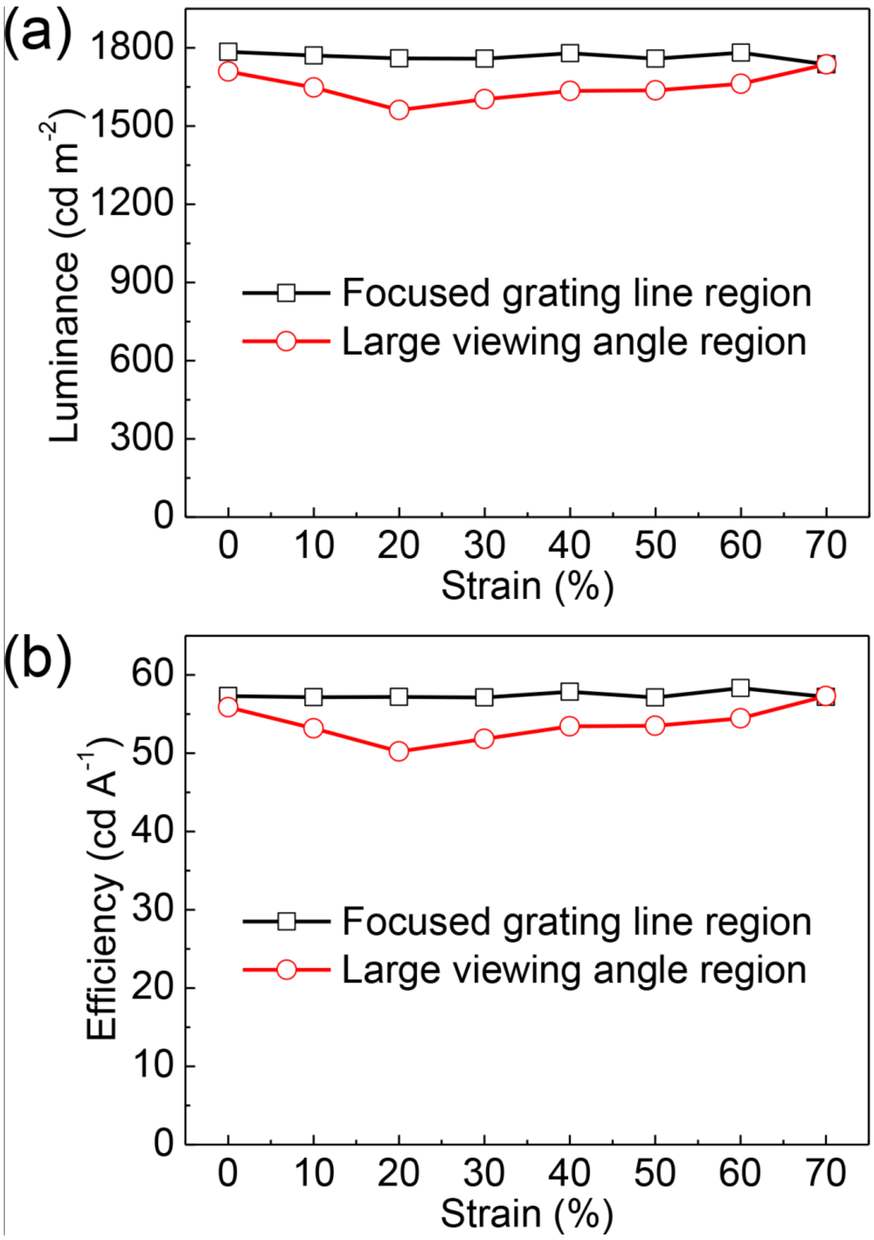

Supplementary Figure 11. EL performance of the stretchable OLED at focused emitting regions. Luminance (a) and efficiency (b) measured from the focused grating line region and the large viewing angle region vs strain values at a driving voltage of 5 V.

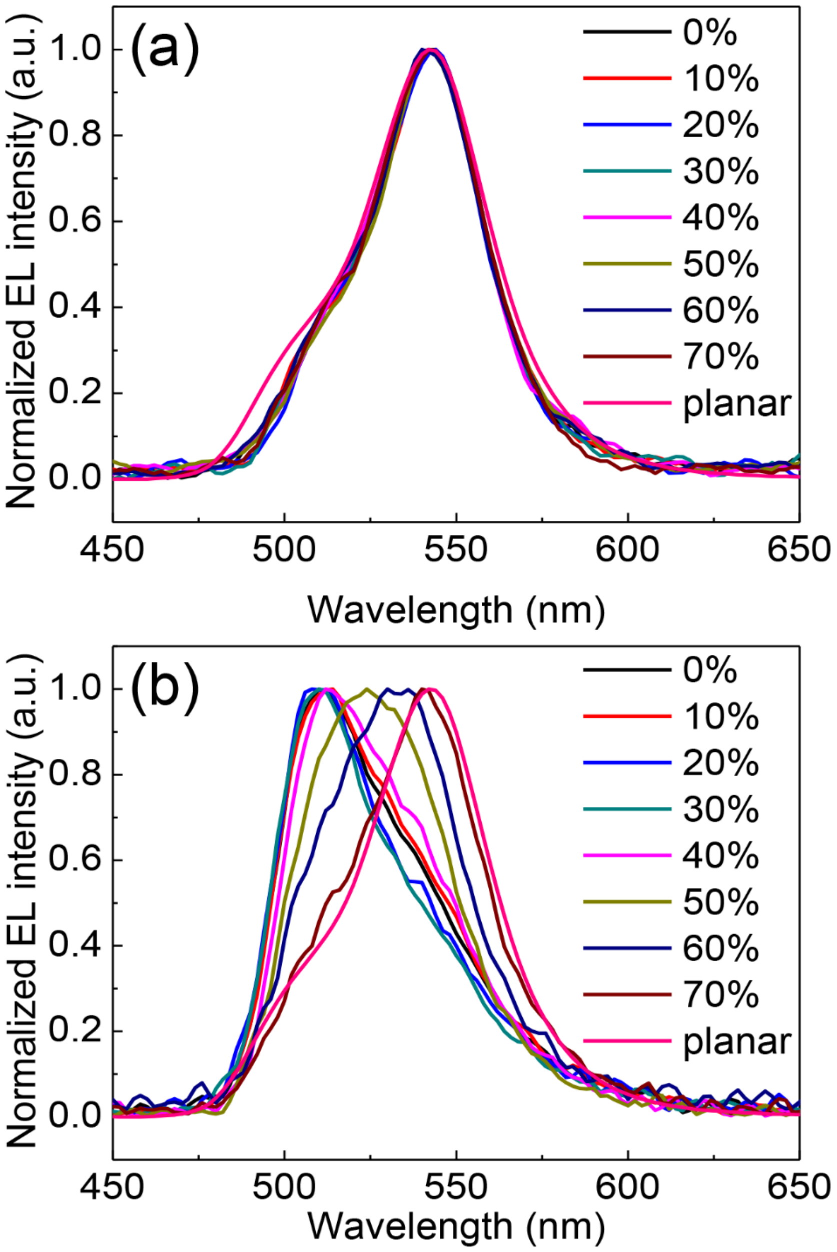

Supplementary Figure 12. EL spectra of the stretchable OLED at focused emitting regions. The normal-directional EL spectra measured from the focused grating line region (a) and the large viewing angle region (b) at 5 V with different strain values. The EL spectra from the focused grating line region are nearly constant due to the nearly invariable morphology at different strain values. While in case of the EL spectra from the large viewing angle region, a microcavity-induced redshift of the peak wavlength can be observed. This is due to the change of the viewing angle for the emission region located at the sharp corner of the buckles. It is nearly 90° at 0% strain and gradually decreases to 0° with tensile strain increasing to 70%.

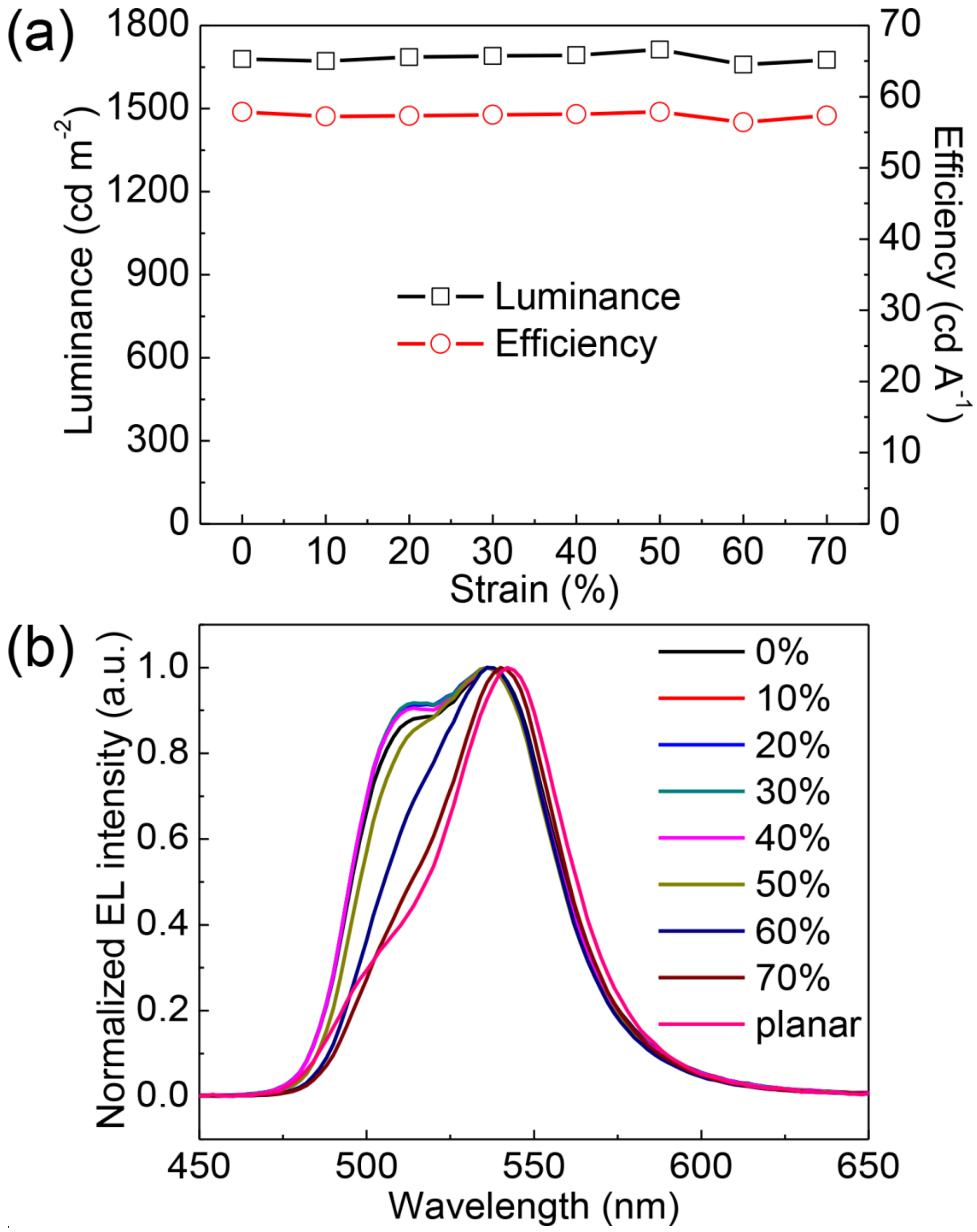

Supplementary Figure 13. Average EL performance of the stretchable OLEDs. Luminance and efficiency (a) and the normal-directional EL spectra (b) measured from a 1350 µm-diameter OLED emitting region at a driving voltage of 5 V with different strain values. Both luminance and efficiency measured from the large emitting region exhibited slight variations at different strain values. The variations are 3.2% and 2.4% for luminance and efficiency respectively. Narrowing and redshift of the peak wavelength of the spectra with increased strain can be observed, which can be attributed to the change of the profile during stretching and similar to that shown in **Supplementary Fig. 9 (b)**.

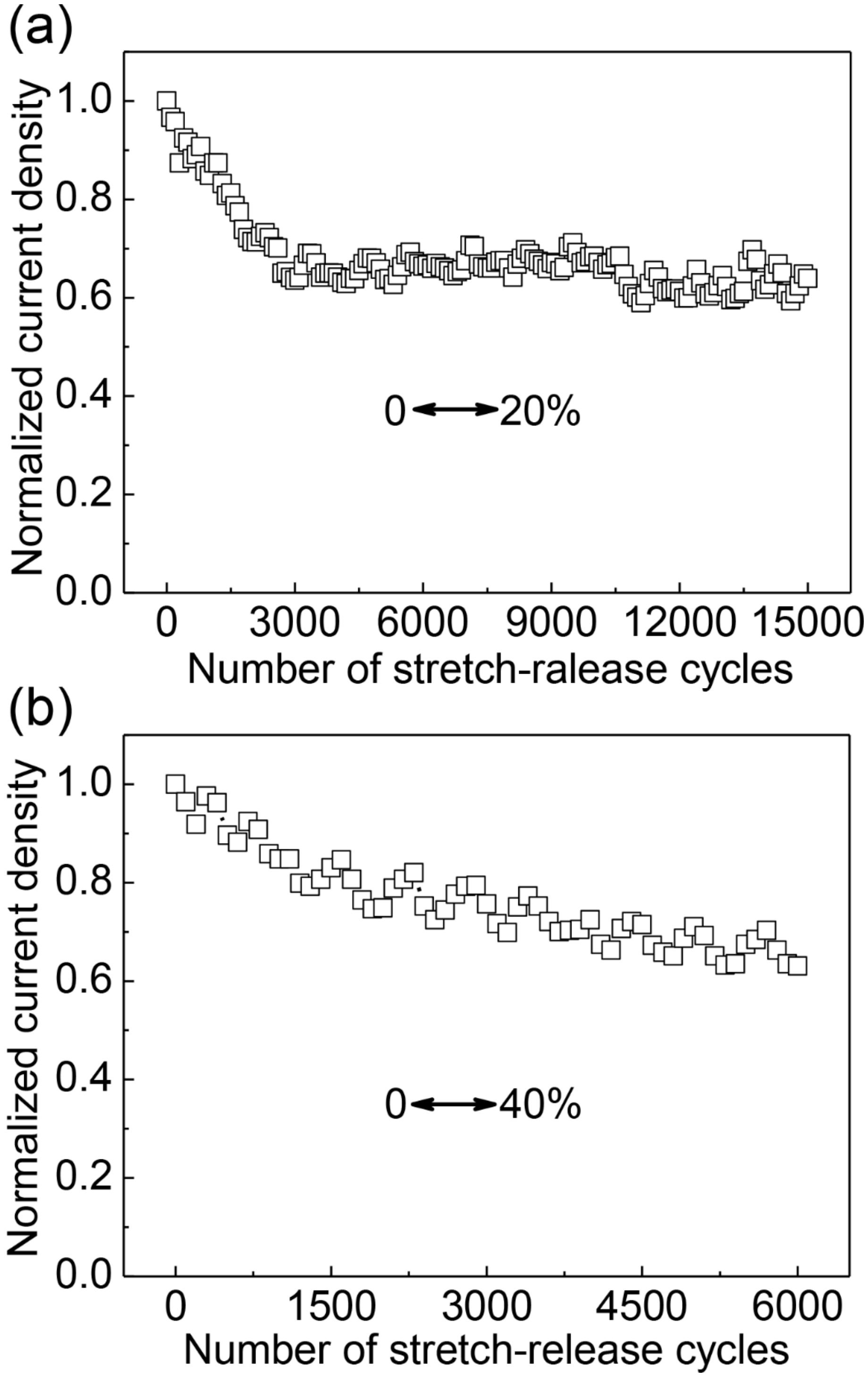

Supplementary Figure 14. Mechanical robust characterization of the stretchable OLEDs. Normalized current density vs the number of stretch-release cycles for the stretchable OLEDs with strains between 0% and 20% (a) and 0% and 40% (b) at 5 V.

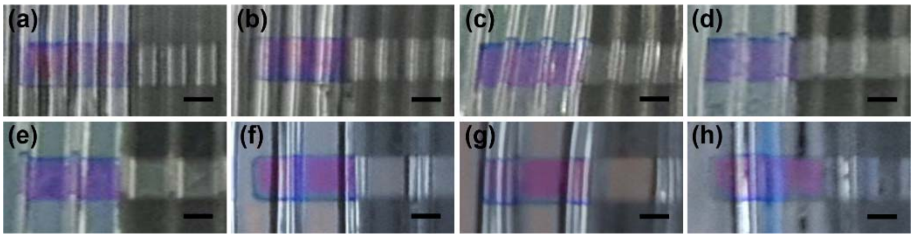

Supplementary Figure 15. (a-d) Photographs of the stretchable OLEDs on the 1-D corrugated elastomeric substrate with a fixed grating line width of 400 µm and increasing grating groove widths of (a) 75 µm, (b) 120 µm, (c) 170 µm and (d) 235 µm. (e-h) Photographs of the stretchable OLEDs on the 1-D corrugated elastomeric substrate with a fixed grating groove width of 170 µm and increasing grating line widths of (e) 600 µm, (f) 800 µm, (g) 1000 µm and (h) 1200 µm. Scale bars, 1cm.

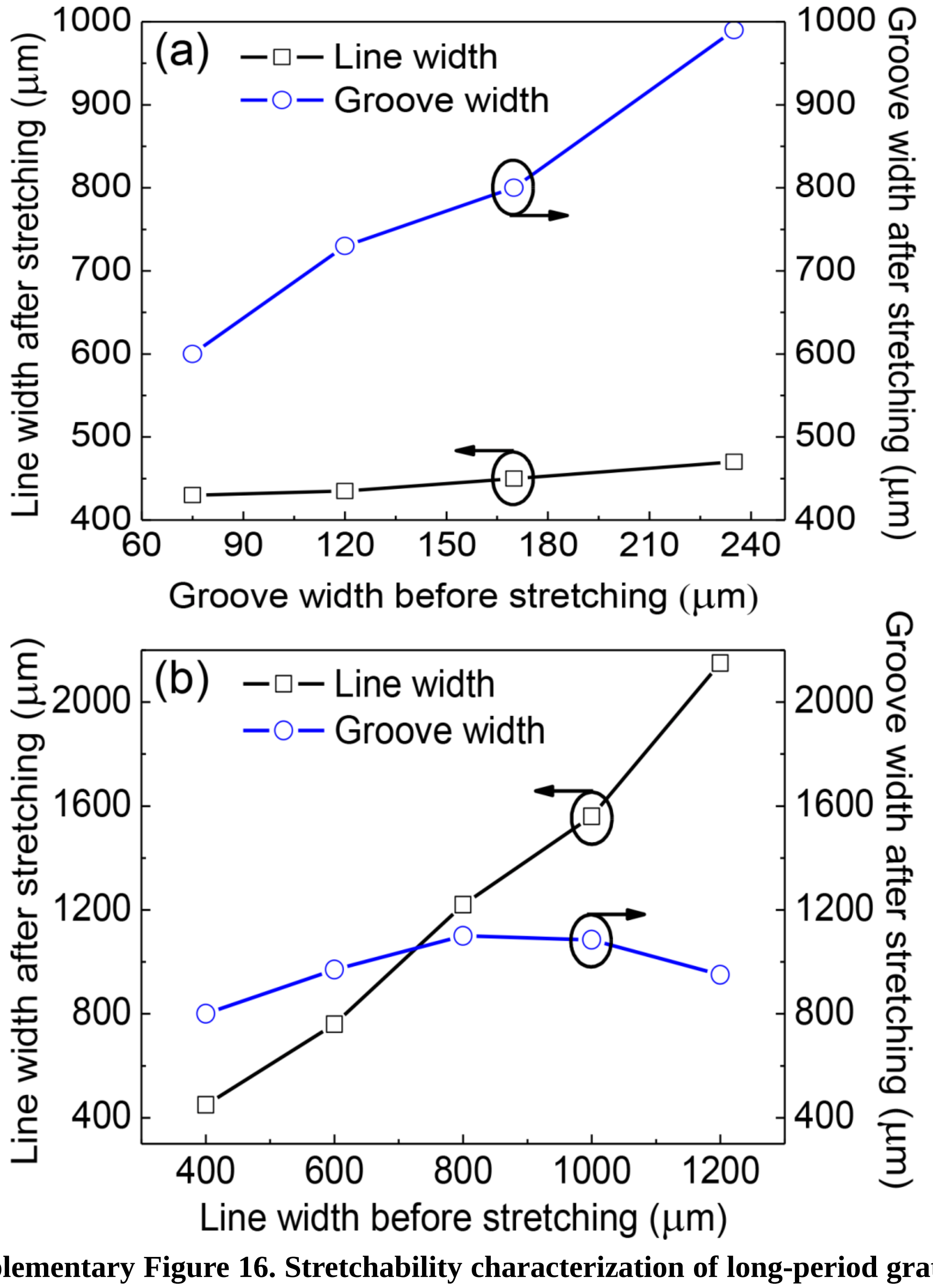

Supplementary Figure 16. Stretchability characterization of long-period gratings. Width of grating line and groove variation after stretching with 120% strain. (a) The width of grating line was fixed at 400 µm and the groove widths were 75 µm, 120 µm, 170 µm and 235 µm, respectively, before the stretching; (b) The groove width was fixed at 170 µm and the widths of the grating line were 400 µm, 600 µm, 800 µm, 1000 µm and 1200 µm, respectively, before the stretching.

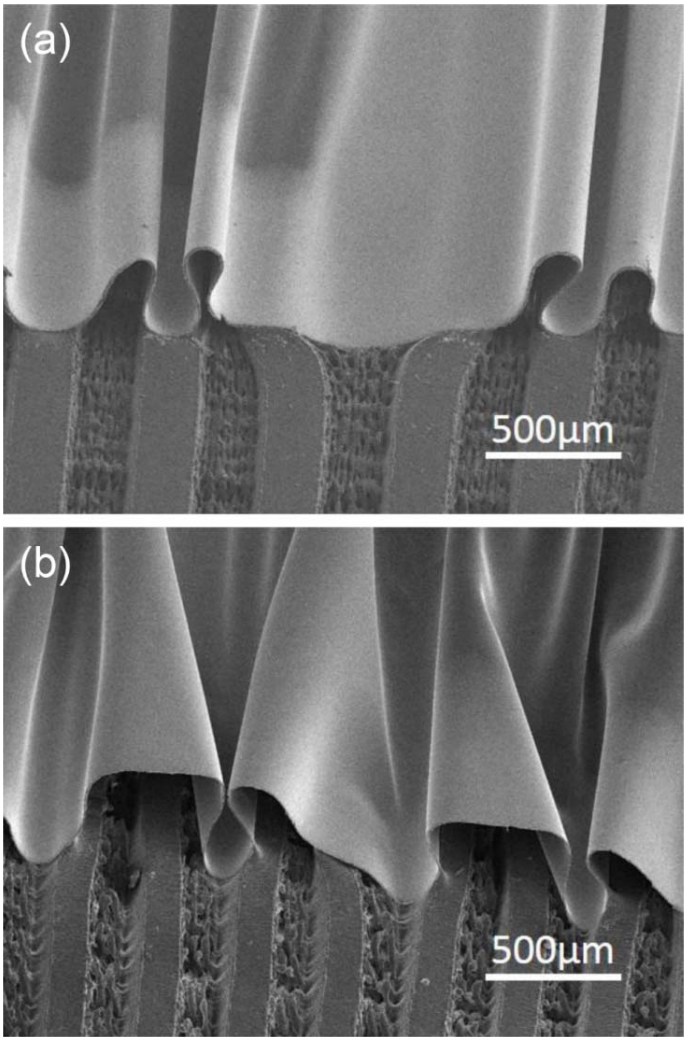

Supplementary Figure 17. Disordered buckling profile. SEM image of stretchable OLEDs with disordered buckling profile. The grating line and groove width of the grating before stretching are 300 µm and 280 µm, respectively (a), and 200 µm and 170 µm, respectively (b).

Supplementary Note 1 - EL spectra of the stretchable OLEDs

Supplementary Fig. 9 shows the EL spectra measured from the OLED region on the grating lines and grooves. Narrowing and redshift of the peak wavelength of the spectra with strain increasing can be observed from both regions, and the spectra at the highest strain of 70% almost coincide with that from the planar device. This phenomenon can be attributed to microcavity effect induced by the top-emitting OLED architecture with metallic top and bottom electrodes. The OLEDs suspended on the grooves have the smallest bending radius at 0% strain, and the bending radius is increased gradually with the increasing of the strain. Larger bending radius of the OLEDs corresponds to smaller offset of the emission direction to the normal direction. The microcavity effect results in a redshift of the emission peak with the decreased offset of the emission direction to the normal direction, which is coincident with the observed redshift of the EL spectra of the stretchable OLEDs with the increased strain. The only difference between the EL spectra from the grating groove region and line region is that the peak intensity at short wavelength from the grating groove region is relative higher compared to that from the grating line region at the small strain values. This difference can be attributed to the smaller bending radius of OLED on the groove, which corresponds to larger offset of the emission direction to the normal direction. Large offset of the emission direction corresponds to a blueshift of the resonant wavelength due to the microcavity effect.

Supplementary Note 2 – EL measurements on both more focused and zoomed-out areas

Photoresearch PR-705 with a tunable lens of MS-55 MacroSpectar was employed for the measurements on both more focused and zoomed-out areas. For the measurements of the focused areas, a 1/8 deg. aperture was selected, which collected light with a field coverage of 120 μm in diameter. For the measurements of average luminance, a 1 deg. aperture was selected, which collected light with a field coverage of 1350 μm in diameter by tuning the MS-55 MacroSpectar lens. They are shown as small (120 μm in diameter) and large (1350 μm in diameter) circles in Supplementary Fig. 10. Here we choose grating line region and sharp corner on the edge of the grating grooves for the focused area measurement. The small circle on the sharp corner of the grating grooves corresponds to the emitting area with large viewing angles to the normal direction. The viewing angle is nearly 90° at 0% strain and gradually decreases to 0° with tensile strain increasing to 70%. Therefore, it is suitable for large viewing angle measurements. While the large circle corresponds to

the field coverage of 1350 µm, which is larger than the largest period of the gratings at 70% tensile strain (about 1250 µm as shown in Supplementary Fig. 4), and is suitable for average luminance measurements.

Supplementary Note 3 – EL performance of the stretchable OLED at focused emitting regions

It can be seen in Supplementary Fig. 11 that the luminance and efficiency measured from the focused grating line region exhibited negligible variations at different strain values. The variations are 2.7% and 2% for luminance and efficiency respectively. This can be attributed to the nearly invariable morphology of the focused grating line region at different strain values. While in case of the measurement from the large viewing angle region, obvious variations in the luminance and efficiency can be observed, which are 10% and 12.4% for luminance and efficiency, respectively. The variations can be attributed to the changed morphology with stretching for the large viewing angle region located at the sharp corner of the buckles.

Supplementary Note 4 –Disordered buckling profile

Supplementary Fig. 17 shows the SEM image of stretchable OLEDs with disordered buckling profile. Increased groove widths result in improved stretchability of the stretchable OLEDs, while, the buckled OLEDs are much liable to falling and adhering on the grooves when the groove widths are too large, which makes the buckles disordered as can be seen in Supplementary Fig. 17a. Decreased line width would also result in improved stretchability. While, the OLEDs were difficult to adhere onto the narrow lines completely and tended to adhere on the grooves, which also leads to disordered buckles (Supplementary Fig. 17b). Therefore appropriated grating parameters are necessary for obtaining ordered buckles and thereafter highly stretchable and mechanically robust OLEDs.
